# Induction of G1 cell cycle arrest and cyclin D1 down-regulation in response to pericarp extract of *Baneh* in human breast cancer T47D cells

**DOI:** 10.1186/2008-2231-20-101

**Published:** 2012-12-28

**Authors:** Parisa Fathi Rezaei, Shamileh Fouladdel, Seyed Mahmood Ghaffari, Gholamreza Amin, Ebrahim Azizi

**Affiliations:** 1 Molecular Research Lab, Department of Pharmacology and Toxicology, Faculty of Pharmacy, Tehran University of Medical Sciences, Tehran, Iran; 2 Department of Microbiology and Molecular cell biology, Faculty of Science, University of Maragheh, Maragheh, Iran; 3 Research Institute for Islamic and Complementary Medicine, Tehran University of Medical Sciences, Tehran, Iran; 4 Department of Biochemistry, IBB, University of Tehran, Tehran, Iran; 5 Department of Pharmacognosy, Faculty of Pharmacy, Tehran University of Medical Sciences, Tehran, Iran

**Keywords:** Pistacia atlantica, Breast carcinoma, Cell cycle, Cyclins, Doxorubicin

## Abstract

**Background and the purpose of the study:**

Natural products from plants have an important role in the development and production of new drugs mainly for cancer therapy. More recently, we have shown that the pericarp methanolic extract of *Pistacia atlantica sub kurdica* (with local name of *Baneh*) as a rich source of active biological components with high antioxidant and radical scavenging activities, has ability to cease proliferation and induce apoptosis in T47D human breast cancer cells. The present study aimed to clarify whether *Baneh* extract able to alter cell cycle progression of T47D cells or not.

**Methods:**

In order to study the possible effect of *Baneh* extract on cell cycle of T47D cells, we evaluated cell cycle distribution and its regulatory proteins by flow cytometry and western blot analysis respectively.

**Results:**

*Baneh* extract induced G_0_/G_1_ cell cycle arrest in conjunction with a marked decrease in expression of cyclin D1 and cdk4 that was strongly dependent on time of exposure. In parallel, Dox-treated T47D cells in early time points were accumulated on S phase, but after 48 h cell cycle progression was inhibited on G_2_/M. Dox promoted striking accumulation of cyclin B1 rapidly and enhanced cyclin A abundance.

**Conclusion:**

Taken together, our results establish that the antitumor activity of the pericarp extract of *Baneh* partly is mediated via cell cycle arrest and downregulation of cyclin D1 and cdk4 expression. These findings warrant further evaluation regarding the mechanism(s) of action of this promising anticancer agent.

## Introduction

Breast cancer is one of the most common cancers in women worldwide and Iran in particular. Each year over 1.15 million women are diagnosed with breast cancer and nearly half of them die [1]. Thus, new therapeutic steps should be taken both to prevent the development of cancer and to lower mortality rates related to it.

The characteristic feature of tumor cells is uncontrolled cell growth as a result of alterations in a variety of molecules and regulatory pathways involved in cell cycle control [2]. Mitogenic signals induce cyclin D1 expression and binding to cdk4/ or cdk6 in G1 phase of the cell cycle. Then cyclin E/cdk2 activated in late G_1_ phase. Cdk2 forms complex with cyclin A in S phase to induce proteins which involved in DNA replication. Cdk1/cyclin A is necessary for initiation of prophase and finally cdk1/cyclin B complex takes part in and completes mitosis [3].

Lately, a significant increase has been seen in the search for new effective cancer chemopreventive and chemotherapeutic agents, particularly for those that have a natural origin and relatively low toxicity [4]. Polyphenolic compounds, in addition to their antioxidant activity, could regulate the genes that are critical for the control of proliferation, cell cycle and apoptosis in cancer cells. Based on epidemiological and preclinical data, polyphenolic phytochemicals possess cancer chemopreventive properties [5]. The cancer preventive effects of polyphenols are due to the regulation of signaling pathways including; nuclear factor-kB (NF-kB), activator protein-1 (AP-1) or mitogen-activated protein kinases (MAPK) [6].

*Pistacia atlantica* sub *kurdica*, known as *Baneh* by the natives, is an Iranian plant from *Anacardiaceae* family; grow in large populations in the western, central and eastern parts of Iran. Its nuts are used by the natives and its gum is used in the production of chewing gum [7]. *Pistacia* species has medicinal applications in different countries [8]. Only phytochemical analysis and apoptosis induction of *Baneh* pericarp extract on T47D and HT29 cells were reported by our group [9,10]. A wide range of investigations have exhibited anticancer potency of mastic gum of *P. lentiscus var. chia* in different cell culture systems including; prostate cancer, colon cancer, human colorectal xenografts, leukemia and Lewis lung carcinoma [9].

In view of the previously mentioned effects of *Baneh* extract on cell viability and apoptosis induction of T47D cells, we investigated whether the *Baneh* extract is involved in cell cycle progression of T47D cells.

## Materials and methods

### Materials

RPMI 1640 and FBS were obtained from Biosera (East Sussex, UK). Pen-strep and Trypsin-EDTA were purchased from Gibco (Paeiley, UK). Doxorubicin (EbeDoxo) was purchased from Ebewe (Unterach, Austria). Methanol was obtained from Merck (Darmstadt, Germany). The cell culture petridishes were obtained from Greiner Bio-one (Frickenhausen, Germany). DAPI (4,6-diamidino-2-phenylindole) and Nonidet P40 were purchased from Roche (Mannheim, Germany).

#### Plant materials and extraction

Fresh unripe fruits from *P. atlantica subsp kurdica* were gathered from Kurdestan province of Iran in June and recognized by Dr. Amin, Department of Pharmacy, Tehran University of Medical Sciences *(6673-THE)*. The pericarp methanolic extract of *Baneh* and its working concentrations were prepared as previously described by our group [9].

#### Cell culture

The human breast cancer cell line T47D (ATCC, HTB-133) was obtained from Pasteur institute (Tehran, Iran) and maintained in RPMI 1640 medium supplemented with 10% heat-inactivated fetal bovine serum, 100 μg/ml streptomycin and 100 U/ml penicillin under conditions of 5% CO_2_ atmosphere at 37°C.

#### Cell cycle analysis

DAPI staining was used to determine the distribution of cells in different phases of the cell cycle by flow cytometry analysis [11]. Briefly, cells were treated with IC_50_ of *Baneh* extract (1 mg/ml) and Dox (250 nM) which was previously determined and reported by our group [9]. Treated and untreated cells were trypsinized, resuspended in DAPI staining solution and analyzed by Partec flow cytometer then data analysis was done using FloMax software.

#### Western blotting

Alteration in cell cycle regulatory proteins in presence of the *Baneh* extract and Dox was investigated by western blot as previously explained [12]. Briefly, the cells were lysed in lysis buffer. Then the total proteins were electrophoresed on a 12% SDS-PAGE, transferred to nitrocellulose membranes (Amersham pharmacia Biotech, Germany) and probed with following primary antibodies: mouse monoclonal cyclin D1 (BD Bioscience, USA); cyclin A, cyclin B1, cyclin E, cdk1, cdk2, cdk4, cdk6 (Santa Cruz Biotechnology, USA) and β-actin (Sigma, Germany); rabbit polyclonal cdk4 (Santa Cruz Biotechnology). We also used goat-anti-mouse IgG and goat-anti-rabbit IgG (Santa Cruz Biotechnology) conjugated to horseradish peroxidase as secondary antibodies. Immunoreactive polypeptides were detected by chemiluminescence using enhanced electrochemiluminescence (ECL) reagents (Amersham bioscience, Germany) and subsequent autoradiography.

#### Statistical analysis

All cell cycle analysis data were shown as mean±SE of three independent experiments. Data were statistically compared using one-way ANOVA with Tukey post hoc and P<0.05 were considered statistically significant.

## Results

### Effects on cell cycle distribution

Flow cytometry method showed that by increasing the time, *Baneh* treated cells were accumulated in G_0_/G_1_ phase compared to the control cells. Within 48 h ~ 80% of the cells were at the G_0_/G_1_ phase versus ~58% in the control cells. Dox exposed cells exhibited accumulation of the cells in S and by increasing the time, the cell cycle pattern was changed to G_2_/M accumulation of the cells and after 48 h 91% of the cells were at the G_2_/M phase against 19% in the control cells (Table 1).

**Table 1:** Alteration in the cell cycle distribution of T47D cells

	**Groups**	**12 h**	**24 h**	**48 h**	**72 h**
	**CTRL**	58.44 ± 0.056	58.74 ± 0.403	58.085 ± 0.96	51.96 ± 0.056
**G**_**0**_**/G**_**1**_	**B**	64.63 ± 0.5^*^	68.25 ± 1.9^**^	79.90 ± 0.91^**^	80.77 ± 31^**^
	**DOX**	36.91 ± 2.63^**^	14.97 ± 0.45^**^	4.59 ± 0.69^**^	3.95 ± 0.11^**^
	**CTRL**	23.35 ± 0.42	25.39 ± 1.86	22.92 ± 2.54	28.45 ± 2.27
**S**	**B**	24.99 ± 1	21.25 ± 0	12.85 ± 1.04^*^	12.17 ± 0.67^**^
	**DOX**	34.71 ± 1.45	49.69 ± 2.55^*^	4.98 ± 0.74^**^	4.51 ± 0.92^**^
	**CTRL**	18.21 ± 0.36	15.86 ± 2.26	18.99 ± 1.57	19.58 ± 2.34
**G**_**2**_**/M**	**B**	10.41 ± 1.45	10.88 ± 0.73	7.24 ± 0.13^*^	7.05 ± 0.36^*^
	**DOX**	27.93 ± 3.46	35.35 ± 2.05^*^	90.9 ± 2.19^**^	91.51 ± 1.03^**^

CTRL: untreated cells.

B: *Baneh* extract-treated cells.

All data were shown as mean ± SE of three independent experiments. Data were statistically compared using one-way ANOVA with Tukey post hoc and *P*<0.05 were considered statistically significant.

*p<0.05 and **p<0.001 in comparison to control RPMI (CTRL).

### Effects on cell cycle regulation

**Figure 1:**
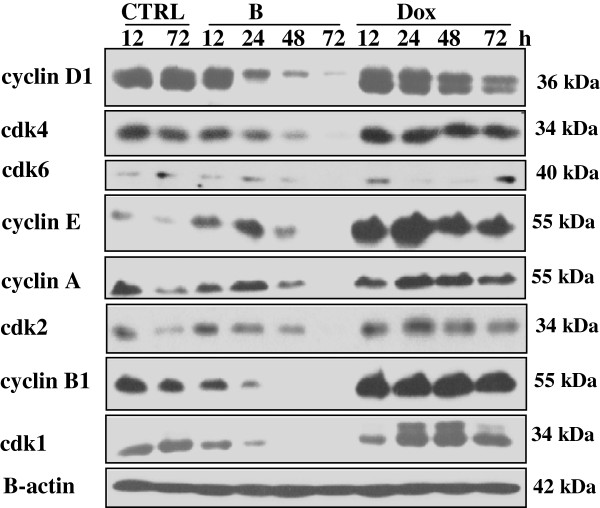
**Expression of cell cycle-regulatory proteins.** Cells were treated with RPMI (CTRL), *Baneh* 1 mg/ml (B) and Dox 250 nM for 12–72 h. Expression of cell cycle-regulatory proteins for indicated time points were analyzed by western blotting. B-actin is showed as loading control. A representative experiment of three independent experiments is shown.

To further ascertain the molecular mechanisms involved in cell cycle block of T47D cells, western blot analysis was performed. As shown in Figure 1, the G_0_/G_1_ block of the cell cycle in *Baneh* treated cells was supported by the intensive down-regulation of cyclin D1 and cdk4, according to the exposure time. Cyclin E protein level was increased at early time points but strong down-regulation of cyclin E was happened after 48 h. Also cyclin A and cdk2 levels were decreased, but not in a time dependent manner, showing considerable decrease at 72 h. In addition, cyclin B1 level was decreased strongly at early time points and approximately disappeared after 48 h. Furthermore, in Dox-exposed cells, the G_2_/M delay of cell cycle progression was confirmed by the induction of cyclin B1 accumulation in comparison to the control cells. Cdk1 protein expression level showed strong increase within 24–48 h in comparison to the control cells. On the other hand, cyclin A and cdk2 levels in Dox exposed cells were higher than control cells. The expression of cdk4 and cdk6 were quietly similar to their expression in control cells. Dox induced upregulation of cyclin E at early time points but its expression was decreased after 48 h. Cyclin D1 expression in *Baneh* treated cells was higher than its expression in control cells within 12 and 24 h but was reduced after 48 h (Figure 1).

## Discussion

Chemotherapeutic approach which uses various natural (plant-derived) agents has become the focus of widespread attention for managing cancers in recent years; it is hoped that such studies will show a positive outcome to provide a scientific basis as to being efficient and useful in chemoprevention/chemotherapy of various cancers.

Reduction in metabolic activity of the cell is due to reduction in number of the cells for cell cycle block and/or cell death. Since the 30% induction of apoptosis in *Baneh*-treated T47D cells could not be enough to explain the reduction in cell viability observed with MTT assay, we decided to check the effect of *Baneh* extract on the cell cycle progression.

Uncontrolled cell cycle has a pivotal role in cancer incidence. Our results demonstrated that *Baneh* extract blocked the cell cycle in G_0_/G_1_ phase in a time dependent manner up to 48 h (Table 1) accompanied by down regulation of positive regulators of G_1_/S transition including cyclin D1 and cdk4, depending on the time of exposure (Figure 1).

With respect to the phytochemical evaluation, the extract possesses considerable amounts of polyphenolic compounds including flavonoids and anthocyanins [10]. Dietary flavonoids inhibit the proliferation of various cancer cells and tumor growth in animal models [5]. Recent studies have shown that polyphenols directly or indirectly can inhibit different cells at different cell cycle phases [6]. Recently, S phase delay of *Baneh-*treated HT29 cells was reported by our group [10]. Pc-3 (prostate cancer cells) treated with gum mastic of *P. lentiscus* showed cyclin D1 downregulation and G1 block of the cell cycle [13]. Treatment of LNcap cells with Gum mastic led to downregulation of cyclin D1 [14]. HCT116-treated cells with chios mastic gum (CMG) were shown G_1_cell cycle arrest [15]. It is established that a single polyphenol is capable of interacting with several protein kinases [16].

Polyphenolic compounds have phytoestrogenic activity [17]. T47D cells are known as ER^+^ (Estrogen Receptor) cells [18]. Estrogenic estradiols, act through the regulation of expression and function of the G_1_ phase regulatory proteins and promote transition through G_1_ phase. Cyclin D1, c-Myc, p21 and cyclin E-cdk2 are estrogen downstream targets [19]. Estradiol can increase cyclin D1 expression by stimulating cyclin D1 transcription via PI3-Kinase/Akt pathway [20]. The inhibitory effect of anti-estrogens on estrogen receptor activity leads to decreased cyclin D1 expression and dissociation of p21 and p27 from the cyclin D1-cdk4/6 complex then association with cyclinE-cdk2 complexes [21]. Because of the promoting effects of estrogen on expression of the cycling D1, it is conceivable that *Baneh* extract involves component(s) which act as anti-estrogenic agent in T47D cells. It has been established that anti-estrogen treatment of MCF7 breast cancer cells induced reduction of c-Myc expression, resulting in cyclin D1 downregulation and eventually cell cycle arrest [19]. In tamoxifen-treated T47D cells, G_0_/G_1_ delay of cell cycle was reported [22]. Cyclin D1 expression and p53 status, both at the gene and protein level, revealed a highly significant association. Cyclin D1 may be one of the downstream effectors of p53 [23]. Loss of G_1_/S control and loss of p53 are two serious factors in tumor formation which directly affect cell-cycle checkpoints [24]. The status of p53 and ER affect the response of breast cancer cells to exogenous agents. T47D cells express mutated and nonfunctional form of p53 [25].

It is reported that NF-κB promotes G_1_-to-S phase transition in mouse embryonic fibroblasts and in T47D carcinoma cells. Inhibition of NF-κB induces pRb phosphorylation and inhibits G_1_-to-S phase transition. NF-κB can functionally interact with other transcription factors which regulate cyclin D1 promoter including; c-Fos/c-Jun, SP1, E2F1 [26]. According to the findings, inhibition of signal transduction proteins in the pathways linked to activation of NF-κB, in part is responsible for the cell cycle arrest and remarkable cyclin D1 reduction induced by *Baneh* extract in T47D cells.

Cyclin D1 is amplified and or overexpressed in a subset of human cancers including breast cancer [27]. Due to the astonishing downregulation effect of *Baneh* extract on cyclin D1 abundance it could be a good candidate for malignancies with deregulated expression of this protein.

Doxorubicin is one of the most common anticancer drugs and is used for the treatment of human cancers. The main anticancer effect of Dox is due to DNA damage and resulting growth arrest or cell death [28]. Dox treated cells shown G_2_/M type of cell cycle arrest associated with cyclin B1enhancment (Table 1 and Figure 1). Increased expression of cyclin B1 and G2/M delay of cell cycle in Dox-treated HT29 cells was reported by our group [10]. Increased cyclin A expression, accumulation of cells at G_2_/M and number of apoptotic cells in Dox-treated k562 cells were described [29]. Maximized level of cyclin B1 protein in T47D exposed cells with Dox (10 ng/ml) has been shown [18]. Previously, Ling et al., demonstrated that in p388 murine leukemia cells, Dox caused G_2_/M cell cycle arrest, reduced p34 kinase activity, increased cyclin B1, alteration of cyclin B1/cdk1 complex function and/or DNA damage may trigger apoptosis [30]. Recently, induction of apoptosis in Dox*-*treated T47D cells was shown by our group [9]. This is the first report to show that *Baneh* extract can cease cell cycle progression of T47D cells.

## Conclusion

Overall, our results clearly indicate that *Baneh* extract as a potent and novel natural anticancer agent able to cease cell cycle progression of T47D cells at G_0_/G_1_ phase associated with downregulation of cyclin D1 by increasing the exposure time. Therefore decreased cyclin D1 expression could be a potential mechanism of growth inhibition by *Baneh* extract. Based on these findings, *Baneh* extract might be a good candidate for cancer therapy but further studies are necessary for in depth investigations of mechanism(s) of action of *Baneh* extract.

## Competing interests

The authors declare that they have no competing interests.

## Authors' contribution

FRP: Conducting experiments and manuscript preparation, FSh: Conducting molecular experiments Ghaffari SM: Supervising experiments. AGh: Supervising herbal experiments AE: Project design, supervising experiments and manuscript preparation. All authors read and approve the final manuscript.
